# The acute effects of a stretching and conditioning exercise protocol for the lower limbs on gait performance- a proof of concept and single-blind study

**DOI:** 10.3389/fspor.2024.1285247

**Published:** 2024-02-08

**Authors:** Felipe B. Santinelli, Aline Prieto Silveira-Ciola, Vinicius C. Moreno, Marina H. Kuroda, Fabio A. Barbieri

**Affiliations:** ^1^ REVAL Rehabilitation Research Center, Faculty of Rehabilitation Sciences, Hasselt University, Hasselt, Belgium; ^2^ Human Movement Research Laboratory (MOVI-LAB), Department of Physical Education, School of Sciences, São Paulo State University (Unesp), Bauru, Brazil

**Keywords:** locomotion, post-activation performance enhancement (PAPE), muscle activity, conditioning exercise, stability

## Abstract

**Background:**

Due to improvement in movement performance, post-activation performance enhancement (PAPE) may open new possibilities to improve gait performance. However, no study has attempted to translate this phenomenon into walking. Therefore, the study aimed to test whether acute stretching followed by a conditioning exercise can improve subsequent gait performance in healthy adults.

**Research question:**

Can an exercise protocol subsequently improve gait performance?

**Methods:**

Sixteen individuals walked four 10-m trials (in each period) before and after 7 min of an exercise protocol composed of stretching (focusing on the lower limb) and a conditioning exercise (standing calf-raise wearing a vest of 20 kg). Gait spatialtemporal parameters and muscle activity of tibialis anterior and gastrocnemius medialis and lateralis muscles were obtained by a 3D-motion system and wireless electromyography, respectively. Before and after the exercise protocol, kinematic and muscle activity parameters were compared by a one-way ANOVA and the Wilcoxon signed-rank test, respectively.

**Results:**

After the exercise protocol, the participants walked with a faster step velocity (*p* < 0.018) and with a lower step duration (*p* < 0.025). Also, higher peak muscle activity (*p* < 0.008) and low-frequency (*p* < 0.034) activation of the anterior tibial muscle after the exercise protocol were observed.

**Significance:**

In conclusion, the protocol improves the stability and the muscles’ efficiency during gait, contributing to a new approach to enhancing gait rehabilitation programs

## Introduction

Post-activation performance enhancement (PAPE), defined as an acute increment of voluntary actions performance following a voluntary conditioning muscle contraction (1, 2), is a phenomenon well-investigated in the sports context. Previous studies have demonstrated improved performance (e.g., increased squat jumps or soccer kicking velocity) after voluntary conditioning contraction or stretching strategies (1, 3). A wide variety of physical approaches, such as dynamic/ballistic (3) or static (4) stretching and conditioning exercises (5), have been used to induce PAPE, suggesting an optimal effect approximately 7 min after (2, 6). The possible mechanisms underlying the PAPE effect are i) increases in muscle temperature, thus improving the functionality of cross-bridge cycling rates; ii) fluid shifts into the working muscles, enhancing muscle function in a fibre type-specific manner (6). However, the studies investigating PAPE on maximizing performance focused mainly on sports context without exploring the impacts on daily activities, such as walking.

Walking is a fundamental mode of human locomotion requiring different neuromuscular, kinematic, and kinetic demands to move forward and maintain stability. For example, gastrocnemius muscles have a primary role during the forward propulsion phase (7). The tibialis anterior (TA) contributes to vertical support stabilising the ankle joint to facilitate forward propulsion (8) and, with the soleus, breaking the forward velocity to heel strike (9). Enhancing the efficiency of lower limb muscles responsible for forwarding propulsion and stability may improve gait performance, as reported in running after a warm-up with a weighted vest (10) and walking after hip muscles static stretching (11). Nevertheless, Radacki et al., (11) only analysed the effects on kinematic gait parameters without investigating muscle activity. Thus, understanding the mechanisms and effects of an acute exercise protocol involving stretching and conditioning exercises on kinematic and muscle activity gait parameters may open new possibilities to improve and apply gait performance in clinical/rehabilitation programs.

In this proof-of-concept study, we aimed to test whether acute stretching followed by a conditioning exercise can improve subsequent gait performance (kinematic and muscle activity) in healthy young adults. We also hypothesize that the exercise protocol would improve gait performance—by enhancing step velocity and muscle activity.

## Methods

### Participants

Sixteen physically active participants (7M/9F, 22.8 ± 0.7 years, 1.68 ± 0.21 m, and 73.2 ± 17.8 kg), according to the American College of Sports Medicine (amount of physical activity: 358.82 ± 63.63 min/week), were recruited. A priori sample size calculation indicated a minimum of 15 participants to detect a significant effect between pre vs. post-conditioning exercise [power of 90% and α = 0.05, gait speed variable- Radacki et al., (2009)]. Participants under 18 or older than 30 years with any neurological, orthopedic, or cardiac disease were not eligible to participate. The participants were requested to avoid vigorous physical activity for 48 h and refrain from caffeine consumption three hours before the testing. The participants provided written informed consent before participating (approved by the University Ethics Committee board- # 53311021.1.0000.5398).

### Gait protocol

**Figure 1:**
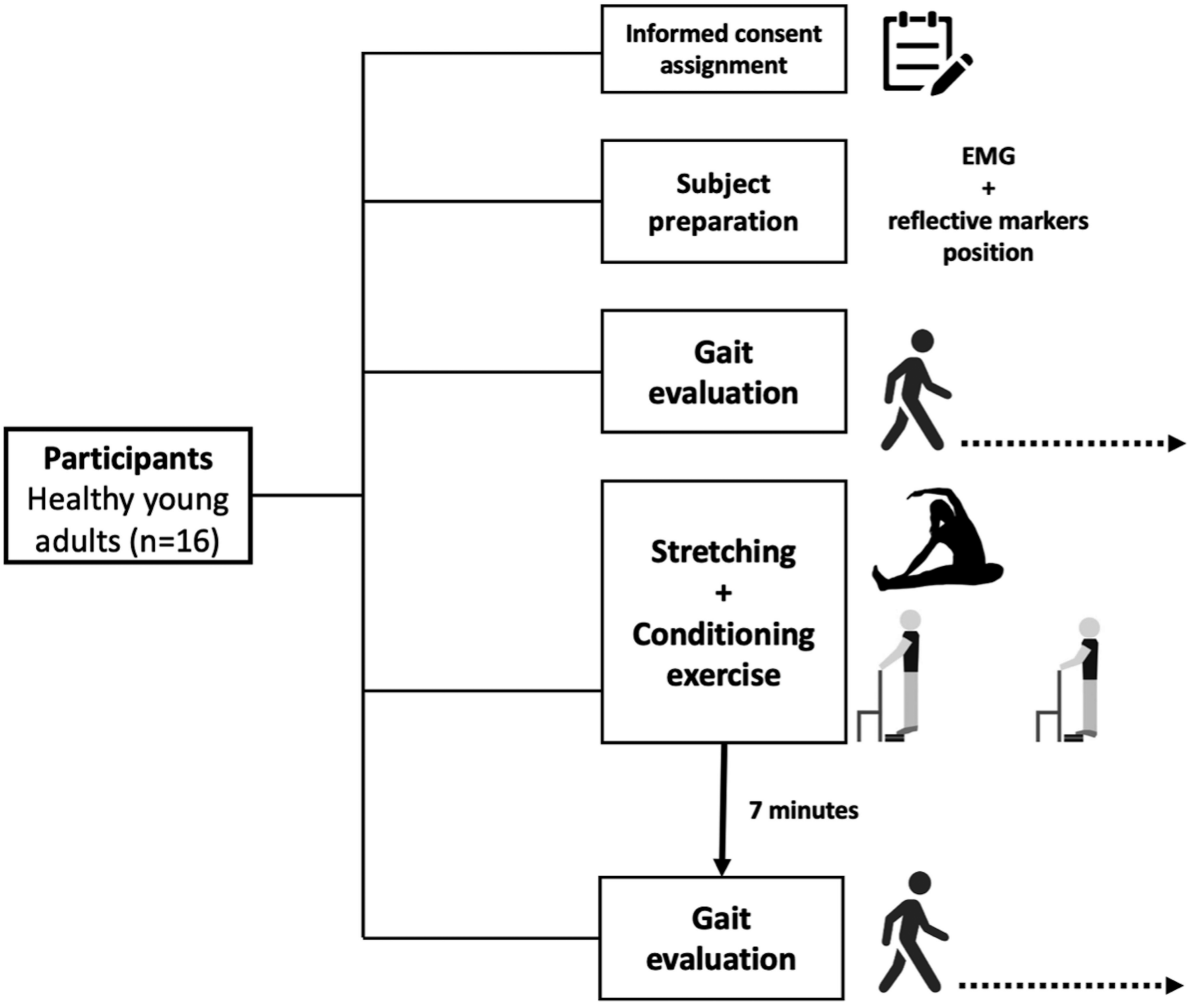
Experimental protocol of the study.

The experimental protocol is presented in Figure 1. Gait was evaluated before and subsequently, after 7 min of recovery, considered the optimal time for PAPE (6, 12) from the exercise protocol. During the recovery, the participants were sitting on a chair. Participants walked in a walkway for four trials each period on their self-selected velocity. A three-dimensional motion capture system (Vicon®, 100 Hz) with ten infrared cameras was used to capture the kinematic parameters. Markers on the 2nd metatarsal and heel, from the left and right feet, were used to calculate kinematic parameters. Synchronized with kinematic data, the EMG activity from both legs of the tibial anterior (TA), gastrocnemius medial (GM), and lateral (GL) muscles were recorded by a Wave Wireless EMG (MiniWave, Cometa System, Italy, 2,000 Hz). The position of the surface electrode followed the recommendation of Surface ElectroMyoGraphy for the Non-Invasive Assessment of Muscles (13).

### Acute exercise protocol: stretching and conditioning exercise

Participants performed a static stretching sequence—two sets of 30s for each focusing on the hamstrings, GL, GM, and TA muscles—followed by a conditioning exercise—four sets of 8 repetitions of a standing calf raise exercise. The standing calf raise exercise was performed while wearing a weighted vest of 20 kg, and the movements were controlled by a metronome (0.5 Hz). At the end of the protocol, the participants were asked how they felt about the effort. For full details, see the Supplementary Material.

### Data analysis

Kinematic data were filtered with a low-pass Butterworth filter of 5th order (zero-lag), with a cut-off of 6 Hz. The average of the five central steps was used to calculate the following parameters: step length, width, duration, and velocity, and double support time (percentage of step duration).

Muscle activity data were filtered using a 4th order band-pass, Butterworth filter (20–500 Hz), and rectified afterward. The muscle activity parameters were individually normalized for each participant according to the maximum value for each variable acquired from each muscle in the gait trials before the acute exercise protocol. To determine heel strike and toe-off in muscle activity signals, we interpolated the kinematic data (20×). Then, in every five steps, we identified two 200 ms windows (1) from 100 ms before to 100 ms after the heel strike (TA activity), and (2) from 200 ms before toe-off (GM and GL activity). These periods depict the propulsion phase (GM and GL) or stability and initial loading absorption (heel strike) stage (TA) during the gait cycle. We calculated the peak value (maximum) of muscle activity and root-mean-square (RMS). We also calculated the low frequency—muscle activity between 25 and 82 Hz, which represents the contractions of type I fibers -, and high-frequency—muscle activity between 142 and 300 Hz, meaning the contractions of type II fibers –, by applying Fast Fourier Transformation (14).

### Statistical analysis

The software SPSS® (V.28) was used for analysis (*p* < 0.05). Shapiro–Wilk, Levene's, and Mauchyly's tests checked data normality, variance, and homogeneity, respectively. An ANOVA for repeated measures with time (before vs. after acute exercise) as the within-subjects factor was used to test for possible differences in gait parameters. For the muscle activity parameters, the variables did not respect assumptions of normality. Therefore, we employed the non-parametric Wilcoxon Signed-Rank test to compare these parameters before and after the acute exercise protocol. The partial eta-square (*η*^2^-small >0.01, moderate >0.06, or large >0.14) and r (small-0.1–0.3: moderate-0.3–0.5: large >0.5) for the ANOVA and Wilcoxon test, respectively, were used as effect size (15). We also calculated and reported (Supplementary Material) the intraclass-correlation-coefficients (ICC) and coefficient of variation (CV) (https://www.socscistatistics.com/descriptive/coefficientvariation/default.aspx).

## Results

**Figure 2:**
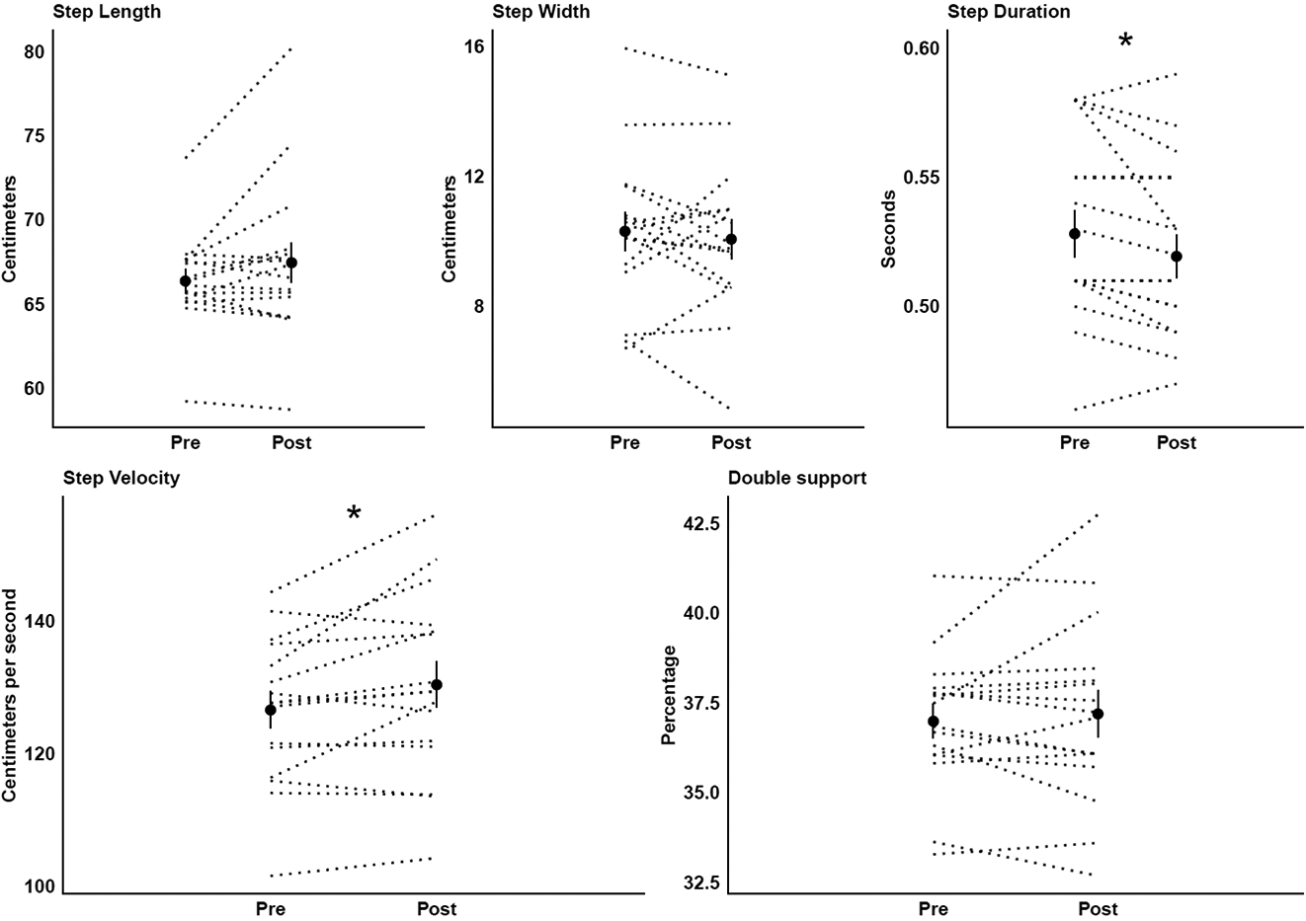
Means (circles) and standard deviations of the kinematic gait parameters before (pre) and after (post) the exercise protocol. Individual data of all participants are represented in dotted lines. * Significant difference between moments.

Participants reported no feeling of tiredness after the exercise protocol. Also, they walked 2.9% (+3.8 cm/s) faster (F_1,15 _= 7.11, *p* < 0.018, *η*^2 ^= 0.32) with 1.7% (−0.01 s) shorter step duration (F_1,15 _= 6.17, *p* < 0.025, *η*^2 ^= 0.29) after vs. before (Figure 2).

**Figure 3:**
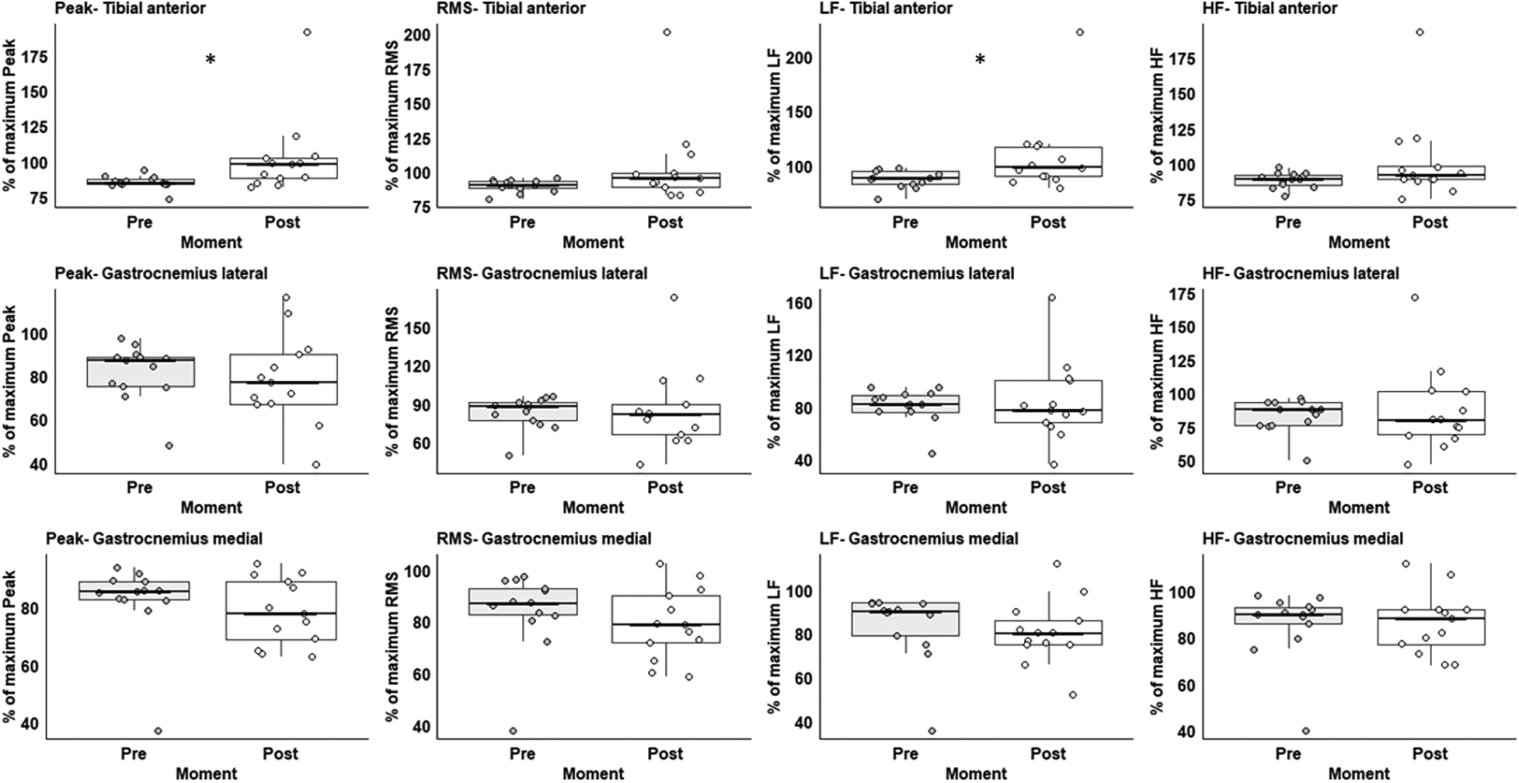
Box plot representation of EMG variables from before (white) and after (grey) preconditioning exercise protocol. Low-frequency (LF), high-frequency (HF), and root-mean-square (RMS). * Significant difference between moments.

Three participants were excluded due to noise for muscle activity. The participants increased 13.3% in TA peak muscle activation (Z = 2.668, *p* < 0.008) and 19.4% recruitments of the TA type I fibre through the low-frequency variable (Z = 2.119, *p* < 0.034) after vs. before the exercise protocol (Figure 3). For the full values of the statistical analysis, please see Supplementary Table S1 in the supplementary material.

## Discussion

Our findings demonstrated that acute static stretching followed by a conditioning exercise (standing calf raise with a fixed load) elicited an enhancement in gait performance and TA muscle activity in young adults. Specifically, we found that after the acute exercise intervention, step velocity and the TA peak muscle activity, and the type I fibers recruitment were increased while step duration was decreased. The PAPE phenomenon could explain the observed enhancement in gait performance and lower limb muscle activity. The mechanism underlying PAPE involves both increases in (i) muscle temperature, which may facilitate the muscle contraction cycle and support an increase in force development (6), and (ii) muscle activity that may increase the efficacy of motoneuron excitability (16), thus improving muscle coordination, and consequently, gait performance. Our study contributes to a new approach to improving gait rehabilitation programs’ efficiency.

Acute static stretching followed by a conditioning exercise improved the stability and the TA muscles’ efficiency during walking. From the perspective of gait stability, a decrease in step duration is associated with a strategy ensuring stability by shortening the unstable phases of the gait cycle (17). In our study, the participants reduced the step duration with a no-significant increase in double leg support time, resulting in a shorter single leg support time. Also, the base of support is smaller in the mediolateral than in the anterior-posterior during walking, which may be a limiting factor in balance control (18). A reduced step duration can facilitate the control of the body's center of mass in the mediolateral (19), mainly when this behavior coincides with an increase in step velocity. Faster walking, which was also observed after a single session of static stretching in older adults (11), decreases mediolateral center-of-mass movements, thus making the gait more stable (20).

From the perspective of muscle efficiency, changes in (higher) TA muscle activity are considered a strategy to keep gait efficiency when gait velocity increases. Specifically, we analyzed the TA muscle activity during early stance, which is a period that TA contracts during ankle plantarflexion to the initial foot contact with the ground (21). Because the amount of leg loading increases with faster walking (22), it is expected an increase in TA muscle activity during this period to control the accelerating and decelerating forces of individual body segments, thus establishing a safe and efficient forward progression (22). Also, increased TA muscle activity is likely critical for interacting with the triceps surae muscles to enhance muscle economy, minimize muscle damage, and contribute to ankle joint stabilization during heel-ground contact (8).

The increased recruitment of TA type I fibers after the exercise protocol is difficult to explain. We can interpret that as TA muscle predominantly presents type I fibers (−69%) (23), it is evident that increased TA muscle activity would cause higher recruitment in this fiber type. Another possible suggestion is related to the characteristics of walking. Despite faster walking velocity requiring higher TA muscle activity, the early stance phase of walking undemand a maximum force production, considering that TA muscle action is isometric during this phase of walking despite muscle-tendon lengthening (21). By acting isometrically, the muscle produces higher forces based on the force-velocity relationship (24), thus storing energy through the elastic properties of the tendon (21). In addition, although the GM/GL has an important role in the propulsion phase of walking, contributing to 34%, the hip joint has a major role in this purpose, contributing to 56% (25). Thus, for not being the major muscle/joint, we were not able to find changes in muscle activation in GM/GL muscles.

These findings presented here are likely to have practical applications. Although acutely, performing stretching in combination with conditioning exercises can improve gait performance, which is essential to prevent falls in many populations. Whether confirmed in populations that suffer from gait impairments, this phenomenon can be an interesting approach and a remarkable intervention strategy to improve gait performance, safety, and stability. However, randomized controlled trials should be done before clinical application.

Although the study showed novelty, some inherent weaknesses and methodological considerations are observed: (i) lack of a control condition (i.e., non-exercising condition) or groups with only exercise and/or only stretching in a randomized, cross-over protocol (exercise vs. no-exercise) on different days. However, this is a proof-of-concept study, involving a small number of subjects and providing the first evidence that an acute stretching in combination with a condition exercise can improve subsequent gait performance. Therefore, we suggest that future studies address this issue; (ii) the experimental design was not double-blinding; (iii) experimental procedures confounders such as diet and hydration were not controlled (6); and (iv) all the participants wore a fixed external load (i.e., the 20 kg vest), not being relativized in accord with their weights. In this way, the muscle activation required and intensity performed were different across subjects. However, although the different effort demands could influence performance, the easy clinical application and the report by the participants that they were not tired after the protocol support our expectations. We recommend that future studies amend these issues when designing the experimental protocol.

## Data Availability

The raw data supporting the conclusions of this article will be made available by the authors, without undue reservation.
