# Supplementary material for: The acute effects of a stretching and conditioning exercise protocol for the lower limbs on gait performance- a proof of concept and single-blind study
Source: Front Sports Act Living. 2024 Feb 8;6:1285247. doi: 10.3389/fspor.2024.1285247 (PMC10881821; doi:10.3389/fspor.2024.1285247)
Supplement: Supplementary file 1 [file Table1.docx]

**Stretching and the conditioning exercise**

The stretching exercise comprised two sets of 30 seconds, with a rest of 30 seconds to each set. Each stretching routine was entirely done before the next one began. In addition, for those stretching exercise that was performed unilaterally, the rest time for one leg was during the time that the other leg was performing the stretching. All the stretching exercise (including active and passive movement) was done close to their maximum perception of tolerance for the individual. For the last, only the conditioning exercise was four sets. The participants accomplished the stretching and conditioning exercise protocol in the following order:

- **Muscle target:** Hamstrings (active movement).
- Position: Sitting on a mat with abduction legs, knees extended, and upright posture.
- Instruction: keep one knee extended with the contralateral limb flexed and with both hands towards the extended leg.


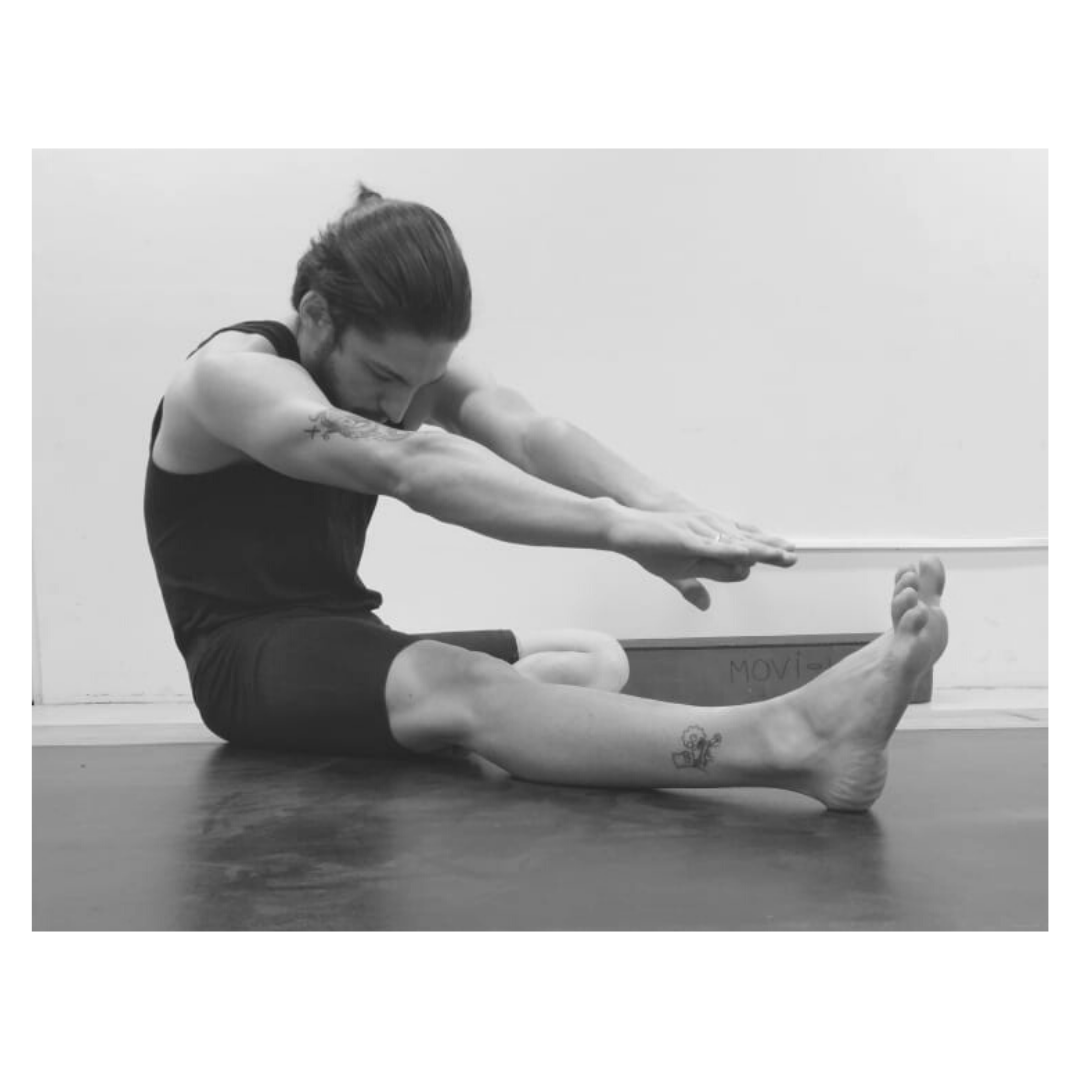


- **Muscle target:** Gastrocnemius (active movement):
- Position: Standing with the forefoot supported on a step.
- Instruction: bilateral stretching; cede the body weight to the hindfoot, performing ankle dorsiflexion and stretching the back of the calf.


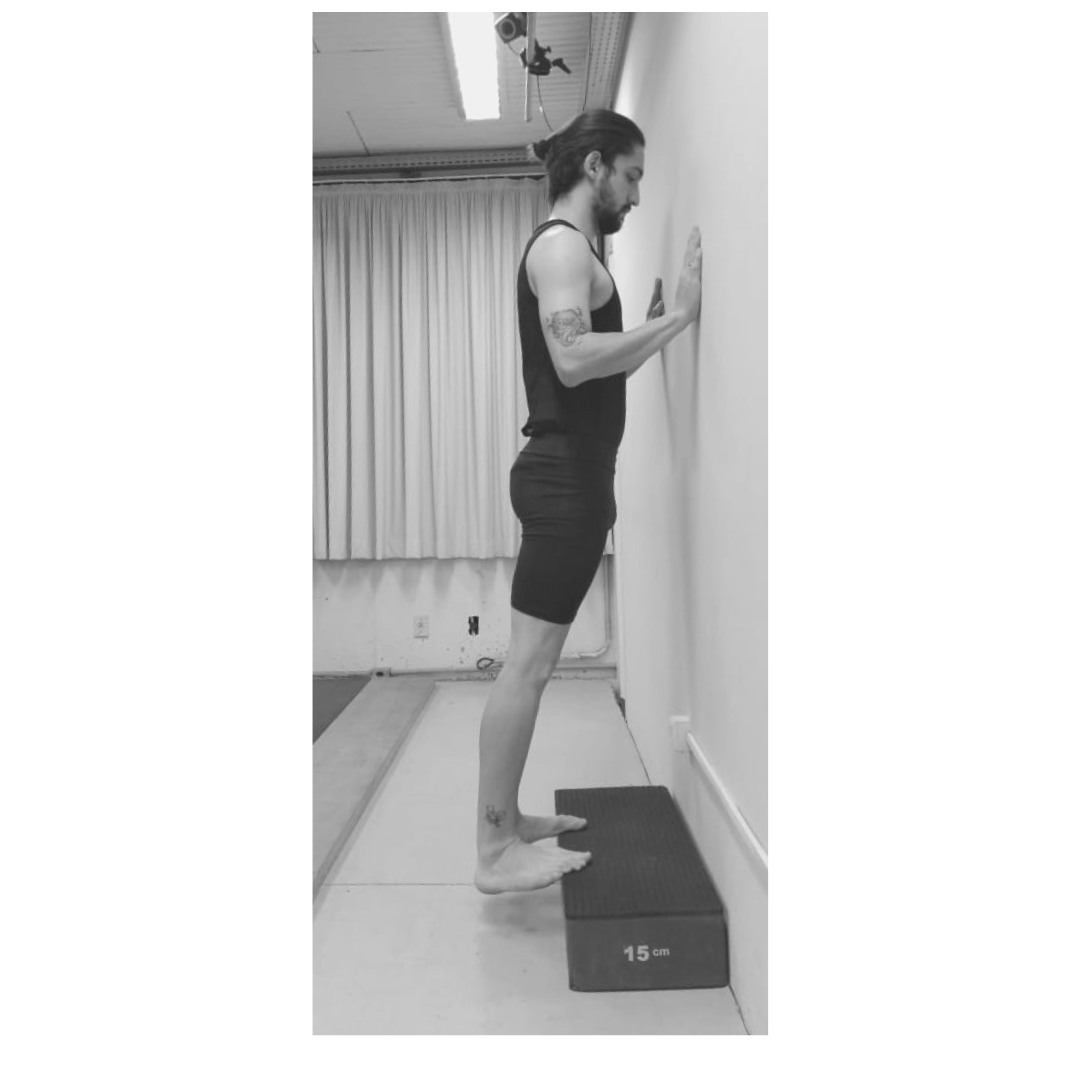


- **Muscle target:** Tibial anterior (passive movement):
- Position: Supine position.
- Instruction: Unilateral stretching; the physiotherapist stabilized the ankle joint and performed a plantarflexion up to the participant's stretch limit and maintained the position. Then he performed the same movement on the contralateral leg.


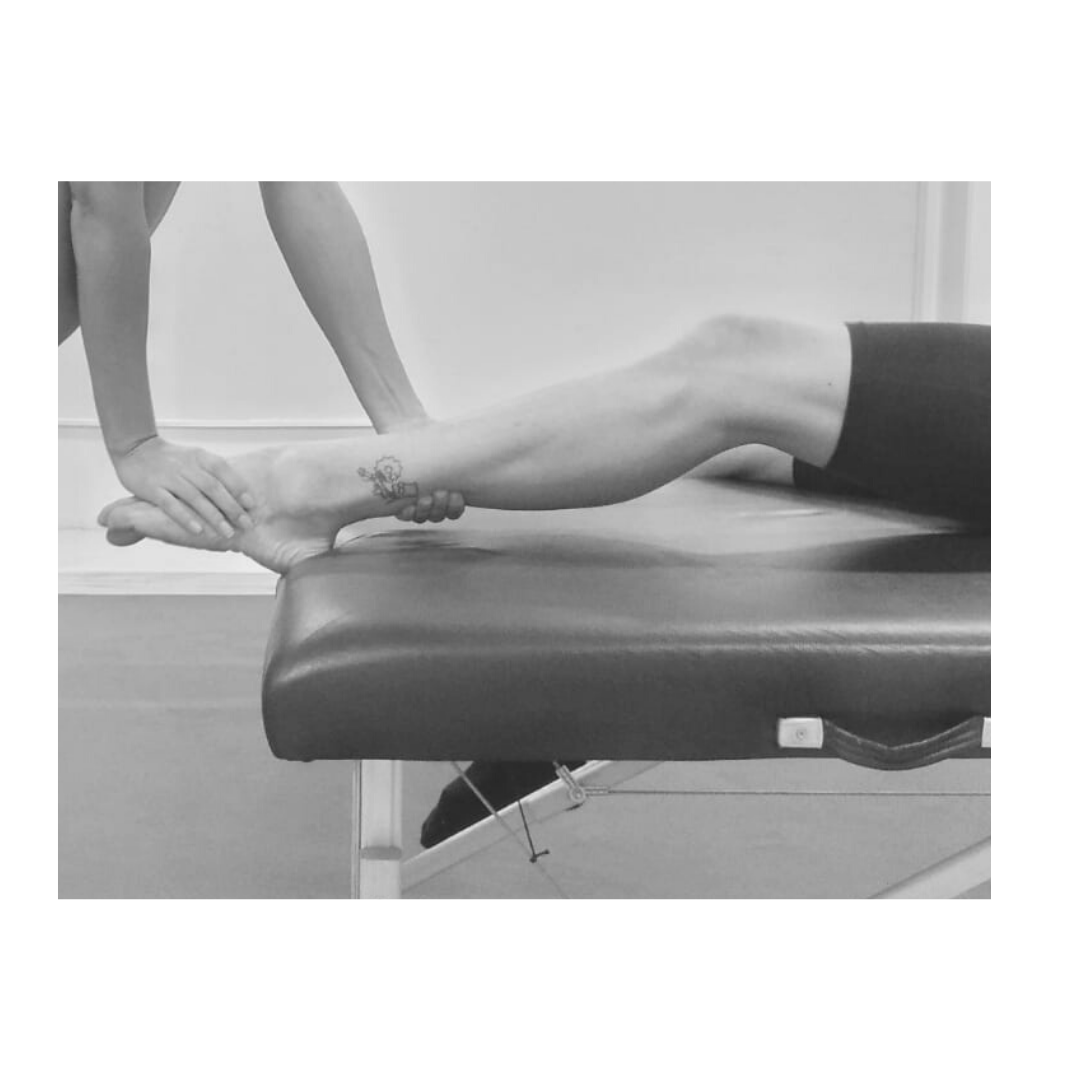


**Conditioning Exercise:** Standing calf raise exercise.

- Four sets of standing calf raise exercise with 8 repetitions, controlled by a metronome (0.5 Hz), in each set with one minute of rest.
- Wearing a 20 kgs vest.
- Maximum movement amplitude.


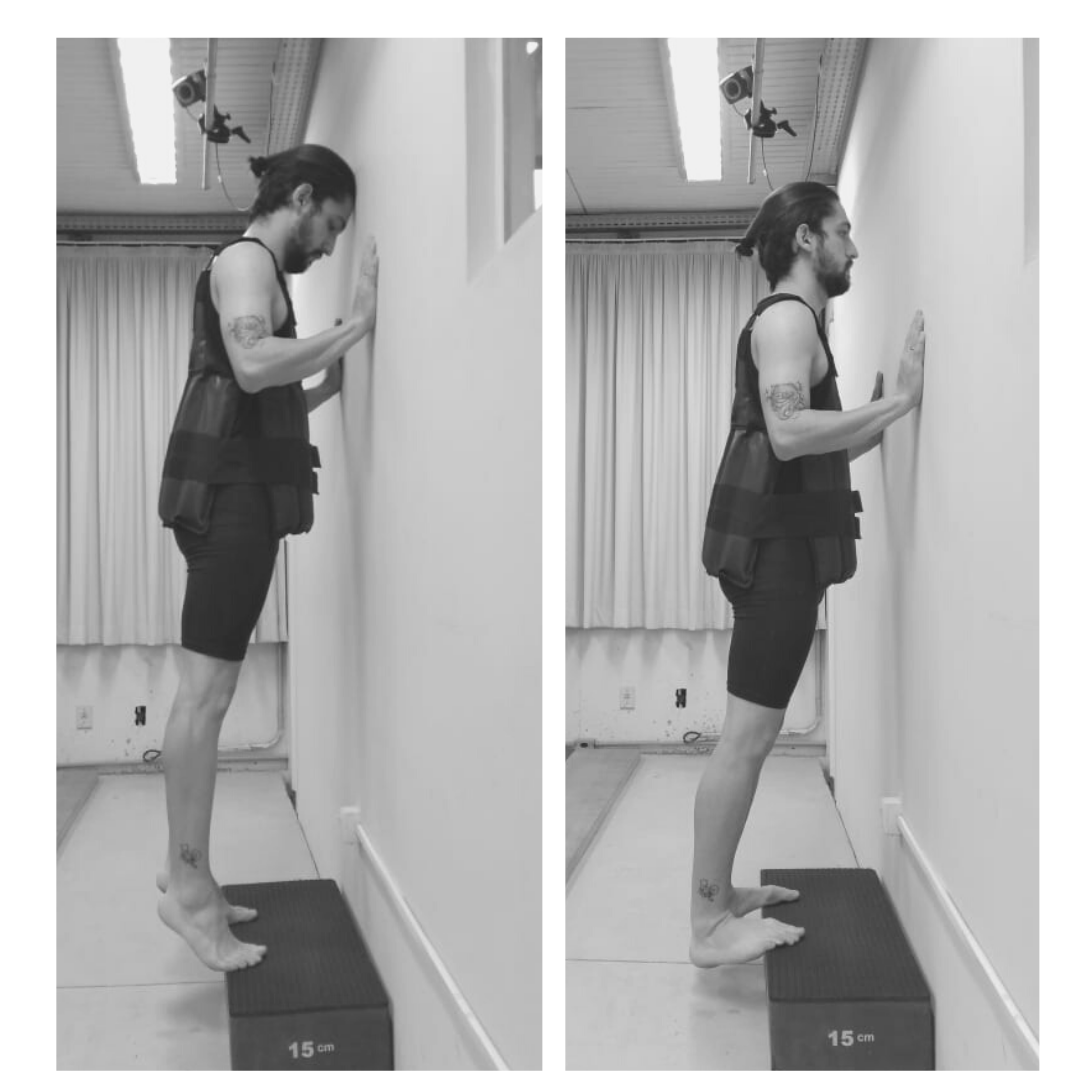


**Table S1.** Statistical values of the Kinematic and electromyograph (EMG) before and after the stretching/conditioning exercise protocol. Intraclass correlation coefficient (ICC), Coefficient of variation (CV). * Significant difference (p<0.05).

| **Kinematic Parameters** | | | | | | | | | | |
| --- | --- | --- | --- | --- | --- | --- | --- | --- | --- | --- |
|  |  |  | **Mean (SD Error)** | **CV(%)** | **F** | **p** | **EF (**η^2^) | **ICC (95% CI)** | **Shapiro-Wilk (p-value)** |  |
| **Step length** |  | **Before** | 66.35 (0.7) | 4.26 | 2.921 | 0.108 | 0.163 | 0.882 (0.663–959)* | 0.008* |  |
|  |  | **After** | 67.45 (1.19) | 7.11 |  |  |  |  | 0.049* |  |
| **Step Width** |  | **Before** | 10.3 (0.59) | 23.01 | 0.432 | 0.521 | 0.028 | 0.895 (0.700–0.963)* | 0.253 |  |
|  |  | **After** | 10.06 (0.59) | 23.84 |  |  |  |  | 0.763 |  |
| **Step Duration** |  | **Before** | 0.52 (0.009) | 6.95 | 6.176 | 0.025* | 0.292 | 0.959 (0.881–0.986)* | 0.05* |  |
|  |  | **After** | 0.51 (0.008) | 6.49 |  |  |  |  | 0.509 |  |
| **Step Velocity** |  | **Before** | 126.52 (2.79) | 8.82 | **7.109** | **0.018*** | **0.322** | 0.946 (0.845–0.981)* | 0.89 |  |
|  |  | **After** | 130.33 (3.47) | 10.63 |  |  |  |  | 0.99 |  |
| **Double Support time** |  | **Before** | 36.98 (0.47) | 5.15 | 0.118 | 0.736 | 0.008 | 0.913 (0.751–0.971)* | 0.535 |  |
|  |  | **After** | 37.18 (0.64) | 6.97 |  |  |  |  | 0.942 |  |
| **EMG Parameters** | | | | | | | | | | |
|  |  |  | **Mean (SD Error)** | **CV** | **Z** | **p** | **EF (r**) | **ICC (95% CI)** | **Shapiro-Wilk** |  |
| **RMS** | **TA** | **Before** | 0.89 (0.126) | 5.07 | 1.260 | 0.208 | 0.247 | 0.124 (-1.31–0.711) | 0.153 |  |
|  |  | **After** | 1.03 (0.086) | 30.16 |  |  |  |  | 0.001* |  |
|  | **GM** | **Before** | 0.84 (0.043) | 18.53 | -1.574 | 0.115 | 0.309 | 0.743 (0.210–0.920)* | 0.001* |  |
|  |  | **After** | 0.79 (0.038) | 17.48 |  |  |  |  | 0.847 |  |
|  | **GL** | **Before** | 0.83 (0.034) | 15.05 | -0.559 | 0.576 | 0.11 | 0.553 (-0.579–0.867) | 0.021* |  |
|  |  | **After** | 0.85 (0.089) | 37.83 |  |  |  |  | 0.032* |  |
| **Peak** | **TA** | **Before** | 0.85 (0.013) | 5.6 | 2.668 | 0.008* | 0.523 | 0.142 (-0.912–0.694) | 0.067 |  |
|  |  | **After** | 1.02 (0.08) | 28.31 |  |  |  |  | 0.001* |  |
|  | **GM** | **Before** | 0.82 (0.039) | 17.28 | -1.334 | 0.182 | 0.262 | 0.674 (-0.021–0.899)* | 0.001* |  |
|  |  | **After** | 0.78 (0.031) | 14.52 |  |  |  |  | 0.272 |  |
|  | **GL** | **Before** | 0.81 (0.036) | 15.86 | -0.804 | 0.421 | 0.165 | 0.746 (0.179–0.922)* | 0.039* |  |
|  |  | **After** | 0.78 (0.057) | 26.89 |  |  |  |  | 0.032* |  |
| **Low Frequency** | **TA** | **Before** | 0.83 (0.022) | 9.45 | 2.119 | 0.034* | 0.415 | 0.018 (-1.342–0.643) | 0.333 |  |
|  |  | **After** | 1.08 (0.101) | 33.86 |  |  |  |  | 0.001* |  |
|  | **GM** | **Before** | 0.83 (0.045) | 19.4 | -0.982 | 0.326 | 0.185 | 0.84 (0.488–0.951)* | 0.001* |  |
|  |  | **After** | 0.80 (0.041) | 18.11 |  |  |  |  | 0.638 |  |
|  | **GL** | **Before** | 0.81 (0.035) | 15.98 | -0.511 | 0.609 | 0.1 | 0.553 (-0.564–0.866) | 0.015* |  |
|  |  | **After** | 0.84 (0.084) | 36.45 |  |  |  |  | 0.122 |  |
| **High Frequency** | **TA** | **Before** | 0.88 (0.014) | 6.04 | 1.364 | 0.173 | -0.267 | 0.122 (-1.369–0.714) | 0.644 |  |
|  |  | **After** | 1.01 (0.083) | 29.71 |  |  |  |  | 0.001* |  |
|  | **GM** | **Before** | 0.86 (0.042) | 17.75 | -0.315 | 0.753 | 0.137 | 0.78 (0.251–0.934)* | 0.001* |  |
|  |  | **After** | 0.86 (0.037) | 15.69 |  |  |  |  | 0.453 |  |
|  | **GL** | **Before** | 0.83 (0.034 | 14.87 | -0.700 | 0.484 | 0.062 | 0.568 (-0.487–0.87) | 0.02* |  |
|  |  | **After** | 0.86 (0.087) | 36.12 |  |  |  |  | 0.042 |  |
